# Consumers' Perspectives on the Design of a New Digital Frailty Education Course, ‘Focus on Frailty’: A Qualitative Co‐Design Study

**DOI:** 10.1111/hex.70287

**Published:** 2025-05-27

**Authors:** Kristiana Ludlow, Benignus Logan, Jhalak Arora, Sarah Martin, Elizabeth Miller, Ruth E. Hubbard, Nicola Warren, Olivia Gallagher, Rosemary Saunders

**Affiliations:** ^1^ Centre for Health Services Research The University of Queensland Brisbane Australia; ^2^ Australian Frailty Network Brisbane Australia; ^3^ School of Psychology The University of Queensland Brisbane Australia; ^4^ Princess Alexandra Hospital Brisbane Australia; ^5^ Medical School The University of Queensland Brisbane Australia; ^6^ Metro South Addiction and Mental Health Services Brisbane Australia; ^7^ Nursing & Midwifery Research Unit, South Metropolitan Health Service Fiona Stanley Hospital Perth Australia; ^8^ The School of Allied Health University of Western Australia Perth Australia; ^9^ Centre for Research in Aged Care, School of Nursing and Midwifery Edith Cowan University Perth Australia

**Keywords:** caregivers, co‐design, consumer engagement, education, eLearning, frailty

## Abstract

**Introduction:**

Frailty‐focused care in hospitals is hindered by systemic barriers, ageism and stereotypes about older adults and frailty. There is a need for frailty education to increase healthcare professionals' and students' understanding of frailty.

**Objective:**

As part of a larger study to co‐design a new digital frailty education course, ‘Focus on Frailty’, this study aimed to explore consumers' and caregivers' perspectives on (i) how frailty and older adults should be represented in frailty education and (ii) what healthcare professionals should be taught about caring for older adults and people who are frail in hospitals.

**Design:**

This was a qualitative co‐design study.

**Setting and Participants:**

Participants (*n* = 25) were older Australians, people living with frailty and family caregivers (collectively, ‘consumers’) who had interacted with the hospital system. This study was conducted in Australia via Zoom and telephone.

**Methods:**

Participants engaged in focus groups or individual interviews and completed a demographic questionnaire and a Research Engagement Feedback Survey. Qualitative data were inductively analysed using template analysis (codebook thematic analysis). Quantitative demographic data were analysed using descriptive statistics.

**Results:**

Seven themes were identified: (1) Consumers' understanding of frailty as loss, deterioration and vulnerability; (2) Utilise a holistic approach to frailty care; (3) Dispel stereotypes; (4) Value consumers' lived experience expertise; (5) Include diverse representation and educate for diversity; (6) Promote meaningful interactions; and (7) Practice care coordination.

**Discussion:**

Participants acknowledged the multifaceted nature of frailty, advocating for holistic frailty education that considers physical, social, emotional, cognitive, financial and spiritual aspects. They described the importance of representing real‐world scenarios and stories, images and videos of real people that reflected the diversity of lived experience. Participants wanted ‘Focus on Frailty’ to include education on individualised care; looking beyond the acute situation; multidisciplinary care coordination that involved informal caregivers; overcoming stereotypes and ageism; and meaningfully interacting with older adults and people who are frail.

**Conclusions:**

Consumers wanted to be represented in frailty education in a way that elevates lived experience and celebrates diversity. They expressed that healthcare professionals should be taught to avoid stereotypes, coordinate multidisciplinary care and engage in meaningful interactions with patients. Consumer‐focused recommendations for designing frailty education were generated.

**Patient or Public Contribution:**

E.M., a consumer partner, contributed to the study design, focus group/interview guide, ethics application and participant information and consent forms. E.M. attended some of the focus groups and contributed to the interpretation of study findings. She also contributed to manuscript revisions. Twenty‐five consumers (family caregivers, older adults and people with lived experience of frailty) participated in focus groups and interviews. Participants shared their perspectives on frailty and contributed to the co‐design of a new digital frailty education course for healthcare professionals and students.

## 1 Introduction

Frailty is a multifaceted risk state characterised by an accumulation of physical, cognitive, social, emotional and functional deficits [1, 2]. People who are frail experience increased vulnerability to acute stressors, including infections, surgeries and medication changes [3]. Self‐reported impacts of frailty include decreased vitality, weakness and fatigue, dependency on others, social isolation and emotional distress [4, 5, 6]. Older adults have described frailty as interchangeable with disablement and a ‘cycle of decline’ [7]. Informal caregivers of people living with frailty (e.g., family members and friends) are also impacted by physical strain, emotional stress, social impacts and financial pressure of care duties [8, 9, 10].

It is estimated that 21% of Australians aged 65 years and older are living with frailty, with an additional 48% being pre‐frail [11, 12]. In a hospital setting, the prevalence of frailty in older people is estimated to be 47% [13]. While associated with chronological age, frailty can also impact younger and middle‐aged adults, with an estimated 27% of younger adults (aged 18–49 years) classified as frail in Australian hospitals [14]. People who are frail have longer hospital stays and recovery time [15, 16], and a higher risk of adverse outcomes such as postoperative complications, in‐hospital falls, delirium, pressure ulcers, disability and inpatient mortality [17, 18, 19].

Frailty‐focused hospital care is hindered by systemic factors such as siloed clinical management, non‐individualised care, staffing shortages and competing demands and priorities [20, 21, 22, 23, 24], contributing to unmet goals of care, unsatisfactory care and recurrent hospitalisation [20, 21, 23, 25]. Ageism can also influence the quality of care and lead to poorer health outcomes for older patients, such as restricted access to treatments and disrespect [26, 27]. While recent research shows that, in general, ageist attitudes in the Australian healthcare workforce are reducing [28], stereotypes and ageist attitudes still persist amongst healthcare professionals and students. Misconceptions and ageist attitudes may, in part, result from limited understanding and awareness of frailty. Healthcare professionals and students report a lack of formal frailty education about understanding, assessing and managing frailty [29, 30]. Arakawa Martins et al. (2020) found that medical students had limited exposure to geriatric medicine or frailty education, and as a result, the majority of participants reported minimal confidence in their abilities to define, assess or manage frailty [30]. Education and exposure to older adults and people who are frail can improve healthcare professionals' and students' perceptions of, and attitudes towards, older adults and frailty [31, 32, 33]. Kelly et al. (2020) found that the use of storytelling through real‐world photographs and videos depicting older adults' lived experiences in an educational resource for Australian nursing students fostered a willingness to engage and connect with older adults and practice person‐centred care, and helped them to overcome communication barriers [32].

Consumer (e.g., patients and caregivers) involvement in the co‐design of healthcare education has gained interest from educators as a way to ‘legitimise’ consumers as experts of their own experiential knowledge and help learners to better understand consumer perspectives [34]. Co‐design involves the meaningful engagement of relevant stakeholders in design processes and decision‐making, facilitating the exploration of lived experiences, perspectives and opinions [35]. It requires active collaboration across research stages, going beyond consultation, to design initiatives that facilitate end‐user engagement and uptake [36]. Research shows that learners report gaining experiential understandings of illness, increased empathy, improved communication and better patient‐centred care when consumers are involved in curriculum design [37, 38, 39]. Involving consumers in the co‐design of geriatric medicine or frailty education is a promising strategy to ensure that education is realistic and engaging and ensures consumer agency [40]. While co‐design has previously been utilised in frailty education with older people and healthcare professionals to develop frailty education for *consumers* [41], Gordon et al.'s (2019) systematic review, which examined the involvement of patients and services users in medical education, found that of 39 studies, no study detailed consumer involvement in geriatric medicine or frailty education for *healthcare professionals or students* [38]. Previous studies have explored older adults' experiences, beliefs and perceptions of frailty more broadly [7, 42, 43, 44]; however, to the best of our knowledge, research has not been published on how older adults and people affected by frailty want to be represented in frailty education.

**Figure 1:**
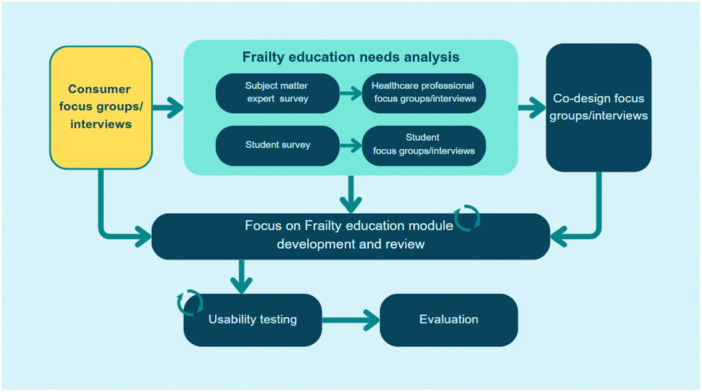
Methodology overview of ‘Development of online learning modules to increase knowledge and understanding of frailty’, highlighting the current study. Regular arrows indicate the order of study phases; circular arrows represent an iterative process of feedback and refinement; the yellow highlighted section indicates the current study phase: consumer focus groups and interviews.

The current study aimed to explore consumers' perspectives on how frailty and older adults should be represented in frailty education and what healthcare professionals should be taught about caring for older adults and people who are frail in hospitals. This study forms part of a larger research project, ‘*Development of online learning modules to increase knowledge and understanding of frailty’* (Figure 1) that aims to co‐design, develop and evaluate a multidisciplinary digital education course comprising education modules on frailty‐focused care in hospitals, called ‘Focus on Frailty’. The course is being co‐designed with researchers, educators, consumers, students and health professionals.

## 2 Methods and Materials

### 2.1 Study Design

This qualitative study involved focus groups and interviews, which provided insights into the experiences, opinions, ideas and perspectives of people with lived experiences of frailty or people caring for someone who is frail [45]. Qualitative methodology enabled rich data collection, resulting in a deep understanding of the research topic [46]. This study was guided by Beyond Sticky Note's mindsets for co‐design [47] and the Consolidated Criteria for Reporting Qualitative Research (COREQ) guidelines (Appendix A).

### 2.2 Ethical Approval

This study was approved by the University of Queensland Human Research Ethics Committee (2023/HE001009). All participants provided informed consent before data collection.

### 2.3 Participants and Recruitment

Individuals were eligible to participate in this study if they were: (a) aged ≥ 65 years or ≥ 55 years if identifying as Aboriginal and/or Torres Strait Islander and had exposure to hospitals (outpatient or inpatient); and/or (b) a caregiver (aged ≥ 18 years) of an older adult who has interacted with the hospital system. Participants were recruited nationally through relevant professional organisations, social media, newsletters and community groups. Potential participants were provided with flyers and study information sheets. Interested individuals contacted the research team via email or by completing an expression of interest form. Purposive sampling was employed to recruit a diverse sample, in terms of location, age and caregiver experience, with efforts to include participants from a variety of ethnic backgrounds. Recruitment ceased when data saturation was reached [48].

### 2.4 Procedures

Participants completed a demographic survey (Appendix B) and engaged in one of five focus groups (comprising 2–5 participants) or an individual interview between July and September 2023. Data were collected via Zoom, and alternative methods of participation were offered where appropriate. One participant engaged in a telephone interview, and no participant selected emailed questions and answers. The semi‐structured focus group/interview guide (Appendix C) was developed through discussion with the research team and reviewed by our consumer partner, E.M. The interviews and focus groups were facilitated by K.L. and B.L., who informed participants of the study goals and activities. K.L. is a health services researcher (PhD) with expertise in co‐design, qualitative research and frailty. B.L. is a geriatrician and PhD candidate with expertise in frailty. E.M., R.S. and J.A. observed some of the focus groups. There were no existing relationships between facilitators and participants.

Focus groups and interviews were video‐ and audio‐recorded. The audio quality of the telephone conversation was too poor to produce a transcript. Instead, detailed field notes were taken of this interview. After each interview or focus group, research team members produced summary field notes outlining any initial impressions of key findings. Participants were invited to complete an anonymous Research Engagement Feedback Survey (Appendix D). Interview participants were given the opportunity to review and provide feedback on their transcripts, with none electing to do so. Focus group participants were unable to do this due to group data aggregation and de‐identification. In accordance with Health Consumers Queensland and the University of Queensland guidelines, participants were offered an AU$45 electronic gift card as remuneration.

### 2.5 Analysis

Data were analysed using inductive template analysis [49], a type of codebook thematic analysis, using NVivo. K.L. and J.A. read through transcripts to familiarise themselves with the data before independently coding 20% of transcripts (*n* = 5). The two researchers then collaboratively identified potential themes and developed a coding template (codebook). The template was applied to all transcripts and was modified throughout the analysis process. K.L. and J.A. then met to devise final themes, which were reviewed by E.M., B.L., R.S., N.W. and O.G. Themes were then compared against field notes by K.L. to ensure key findings were captured.

## 3 Results

Twenty‐five people participated in this study (17 focus group participants and 8 interview participants). Focus groups ranged from 38 to 72 min (mean = 58 min), and interviews ranged from 33 to 65 min (mean = 45 min). Most participants were female (64%), aged 71–80 years old (48%) and living in a suburban area (60%) (Table 1). Ten participants (40%) had a caregiving role for an older adult or someone living with frailty. Four participants (20%) aged 65 years or older also identified as being a caregiver for an older adult or someone who was frail.

**Table 1:** Participant demographic characteristics.

Age range (years)	*n* (%)
30–40	2 (8.0)
41–50	0 (0.0)
51–60	3 (12.0)
61–70	6 (24.0)
71–80	12 (48.0)
81–90	2 (8.0)
Gender	
Female	16 (64.0)
Male	9 (36.0)
Prefer not to say	(0)
Self‐described gender	(0)
State/Territory	
Australian Capital Territory	2 (8.0)
New South Wales	3 (12.0)
Northern Territory	0 (0)
Queensland	5 (20.0)
South Australia	2 (8.0)
Tasmania	2 (8.0)
Victoria	8 (32.0)
Western Australia	3 (12.0)
Location	
Metropolitan: Inner city	5 (20.0)
Metropolitan: Suburban	15 (60.0)
Rural or remote (including regional centres)	5 (20.0)
Caregiver	
Yes	10 (40.0)
No	15 (60.0)
Ethnicity*	
Australian**	11 (44)
Caucasian***	7 (28)
Scottish	1 (4)
Anglo	1 (4)
Dutch	1 (4)
Greek	1 (4)
Indian	1 (4)
South Asian	1 (4)
Sri Lankan/born in Singapore	1 (4)

* Ethnicity was captured via free‐text responses.

** Four participants included a classifier: ‘White’, ‘Anglo’, ‘European’ and ‘White—born in South Africa’.

*** Two participants included a classifier: ‘Dutch’ and ‘French/Ukrainian/American’.

### 3.1 Themes

Seven themes were identified (Table 2). The first theme reflected participants understanding and perceptions of frailty. The other six themes represented participants' ideas and recommendations for Focus on Frailty's design and content.

**Table 2:** Overview of themes.

Themes	Description
Consumers' understanding of frailty as loss, deterioration and vulnerability	Participants associated frailty with loss and deterioration, as well as vulnerability and risk. Frailty was seen as a consequence of a gradual decline in health, with some participants noting a frailty ‘turning point’ brought on by a change in circumstances.
Utilise a holistic approach to frailty care	Participants recognised that frailty was influenced by physical, cognitive, emotional, mental, social, spiritual and financial aspects of life. They advocated for a holistic approach to care, which included looking beyond the hospital setting to ensure patients had support post‐discharge.
Dispel stereotypes	Participants said that healthcare professionals should be taught not to make assumptions about patients based on their age, frailty status or the way they looked. They acknowledged that frailty looked different for different people and therefore healthcare professionals should not make generalisations about patients.
Value consumers' lived experience expertise	Consumers were recognised as experts in their own health, with knowledge that should be valued by healthcare professionals. Participants wanted patients' real‐life stories to be featured in the education course.
Include diverse representation and educate for diversity	Participants wanted to see diversity of experience captured in the education modules in terms of age, gender and sexuality, culture and ethnicity, and experiences. They also saw a need for healthcare professionals to be educated on interacting with patients from minority groups.
Promote meaningful interactions	Participants advised that the education course should teach healthcare professionals how to have meaningful interactions with patients, including getting to know patients and providing individualised care; adapting communication to individuals' needs; actively listening; building rapport; creating trusting environments where patients felt comfortable; taking time with patients; and treating patients with respect.
Practice care coordination	Participants noted that frailty involved different disciplines and advocated for multidisciplinary teams, peer workers* and social workers to coordinate care. They also recognised the importance of caregivers in providing care, sharing knowledge and making decisions.

* A peer worker, or a peer support worker, is someone who applies their lived experience to provide emotional, social and practical care.

#### 3.1.1 Consumers' Understanding of Frailty as Loss, Deterioration and Vulnerability

Participants spoke about frailty in terms of loss, decline and deterioration. Frailty was often described in comparison to a past, more ‘robust’ or ‘fit’, health state. Participants acknowledged that frailty meant a loss of function or the inability to complete daily living tasks, leading to a loss of independence and a greater need for more support.

‘*I'm not as able to run around like I used to, you know. So, there's more fatigue and so forth. I walk slowly. It takes longer for the brain to figure out that I've just done a step so I have to take longer time to walk down the stairs and make certain that that I give time for my brain to figure out that I'm at the next step.’* (P20)

Participants associated frailty with weaknesses and slowing down. While this was often in relation to physical ability, for example, mobility and strength, participants acknowledged that weakness could refer to cognitive, emotional and social aspects (see Theme 2).

‘Sometimes *it's mentally, it's socially … physically. But yes, your standard that you were used to obtaining is slightly dwindling away … I just see it as a gradual slowing down of absolutely everything.’* ^1^ (P6)

Participants alluded to the dynamic nature of frailty, explaining that declines often occurred gradually. Some participants referred to a point in time where people became frail, a tipping point, often as a result of a change in life circumstances, for example, loss of a job, partner or a pet or a change in financial situation.

‘*And that there are a number of tipping points, if you like, such as a loss of a carer, or a mate or a pet even.’* (P10)

Some participants recognised that frailty resulted from changes over time, and therefore, things could be done to slow down its progression and, in some instances, reverse it. They spoke about the importance of lifestyle factors (e.g., exercise and nutrition) and medication optimisation, as ways to prevent and manage frailty.

‘*… for example, it's more painful to walk around so they do less. You want to do a bit more, it becomes harder. But you should take up that challenge, and the same with the eating and you know, look at your diet and concentrate on that and you can make a difference, and you can either slow it [frailty] down or even reverse it.’* (P3)

The words ‘vulnerable’ and ‘vulnerability’ were used to describe people who are frail. Participants explained that people who were frail were at a greater risk of illness and injury, particularly falls. Some recognised that frailty was accompanied by a loss of confidence, and healthcare professionals needed to help patients build their confidence and change their mindset.

‘*I feel I used to think that it entails extremely limited mobility. But more and more I've come to realize that it's also very much tied to a lack of confidence in movement. And so, there is that certain mental health aspect to it.’* (P24)

While participants tended to associate frailty with ageing and older adults, many recognised that frailty falls along a spectrum and people of different ages can be frail.

‘*We think of when we hear the word frailty, we think of older people. Younger people can actually go through that.’* (P20)

#### 3.1.2 Utilise a Holistic Approach to Frailty Care

Many participants shared the need for a holistic approach to frailty care, recognising how the different facets of a person's life can influence frailty. The most discussed domains were physical, cognitive, social, emotional and mental health. Less commonly identified domains were spiritual and financial. Participants spoke about the interconnectedness of these domains and the need to look at the whole person.

‘*But definitely, it's [frailty] a holistic thing.’* (P17)

‘*… looking at where I sit from a mental aspect, from a physical aspect, from an emotional aspect, social, spiritual, and financial aspect … It's [frailty is] more than just the physical capacity of an individual. It's also looking at their emotional state, their social state.’* (P21)

A holistic approach to frailty also meant that healthcare professionals needed to consider the patient journey and patients' lives beyond the hospital. One participant suggested that hospitals concentrated on acute illness, which was not conducive to frailty‐focused care:

‘*My experience in hospitals is that the health professionals focus on the acute problem and that is what places frail people often at very great risk, because as a general rule, someone who is frail has at least one, or if not more, disabling aspects to their frailty and also commonly have chronic illnesses and hospitals … don't deal with managing the chronic illnesses and they overlook the disabilities.’* (P9)

Participants expressed that healthcare professionals needed to think about patients' home environment, access to resources and post‐discharge support. A few participants extended this consideration to caregiver support.

‘*And far too often I've been around people who've been discharged to home when I can't even get themselves to the toilet … So, I think, discharge, and that … awareness of what are you releasing a person to, it's pretty critical content.’* (P9)

#### 3.1.3 Dispel Stereotypes

There was an overarching message throughout focus groups and interviews that healthcare professionals should be taught not to make assumptions about someone based on their age, the way they look or because they are frail.

‘*I think it's really important for students, whether it's doctors, nurses, psychiatrists, allied health, whoever is coming into a hospital setting, to really have ingrained in them from day one, you do not make a judgment call on the way that someone looks.’* (P12)

The sentiment of not judging someone by the way they look was more commonly discussed in relation to older adults, for example, healthcare professionals assuming that older adults need to use a ‘walker’ for ambulatory assistance or that they can't hear well.

‘*When I've been visiting people in hospital, and they are elderly, is the thinking that they [healthcare professionals] need to speak slowly and loudly. When in my case, my hearing is okay. So, it's a perception … so just jumping to conclusions and making those assumptions.’* (P18)

Conversely, a couple of participants explained that frailty could be missed if someone looked healthy:

‘*Some people may look all right, but they're actually not; they don't display normal symptoms of this frailty. So, I think the assumption is there that they need to be probably mindful of that, that someone might look all right but they actually behind the scenes … they aren't all right.’* (P6)

Common misconceptions about older adults reported by participants included older adults not having agency, not being able to understand what is being said and not being able to communicate or express themselves.

‘*I think what I find is a common misconception is that because you're frail you don't have a sense of agency, that people can talk about you and for you and around you and sometimes, when a person is very frail, it might be difficult to communicate with them. But always that person is still with us, and it's still important and central in their own life. So, I think that sense of agency.’* (P9)

‘*Just because you're frail doesn't mean that you don't have a voice.’* (P25)

In some instances, misconceptions were said to lead to inappropriate advice or treatment.

‘*I was disgusted by the way my grandmother was treated in the gen med [General medicine] ward*—*[when translating information from a healthcare professional to P22's Italian grandmother]*—*I'm like, “I wanna make sure that she's understanding.” And the guy said, “but she's demented.” And they said this in front of her and … it took everything I had to not give him a good talking to … she hasn't lost all of her decision‐making capacity. She still has her personality. She's still verbal. She still interacts. And there was just this assumption—while she has dementia, therefore she has no capacity.’* (P22)

Few participants discussed terminology and language, but those who did said that terminology in the education course should be person‐centred (e.g., ‘a person who is frail’ as opposed to ‘a frail person’) and should avoid perpetuating stereotypes.

#### 3.1.4 Value Consumers' Lived Experience Expertise

Participants explained that people are experts of their own health, bodies and experiences. They alluded to healthcare professionals not recognising patients' expertise when they should instead be valuing their knowledge.

‘*… one person … could almost in a sense … have a PhD in their own illness, but they don't have the medical degree or other health professional degree, but they have that that knowledge there. And if that's not accepted and honoured, then that can lead to a lot of miscommunication occurring, and the health professionals that I've come across seem to have a very difficult time trying to get this idea of lived experiences.’* (P10)

Participants emphasised that patients' lived experience should be showcased throughout Focus on Frailty. They wanted the course to include real stories and feature real people in images and videos. Storytelling was described as a powerful method for enhancing learning. Some participants had a negative reaction to cartoons and animations representing patients, advocating for photographs instead.

‘*But I know many, many, many people who would be so happy to have their stories represented. And so often, when you use, say, stock photographs for a model as opposed to someone who is genuinely frail, you're misrepresenting to some extent and so I'd be really inclined to encourage you to pursue genuine case studies as opposed to yeah, invented examples … you'll find real people that you can do that with and it makes it more vivid. That's my reason for not liking the cartoons, because it de‐personalizes it. And I think if you want … the people you're training to really take it on board, then you need to appeal to their humanity. And you do that through real people.’* (P5)

One participant noted the importance of meaningfully involving people with lived experience (consumers) in the design of Focus on Frailty:

‘*I just want to say, thank you for the opportunity. It's reassuring … that people like yourselves are listening to the people with living and lived experience. Because that's what true co‐concept/co‐design is about … it's not just that tokenistic—we'll bring you in at the end just to give it that rubber tick—so thank you for leading the way.’* (P21)

#### 3.1.5 Include Diverse Representation and Educate for Diversity

Participants emphasised that case studies, videos and images within ‘Focus on Frailty’ needed to showcase diversity in terms of consumers and their experiences. Participants said that the course should feature people from different cultural and ethnic backgrounds, different genders and the LGBTQIA+ community. Participants advocated for Focus on Frailty to be inclusive.

‘*… it's all about, you know, inclusion, and also about showcasing the diversity of the people you'll be representing, whether it's someone in a hijab or someone in a wheelchair, but don't make it so stereotypical, having an old person in a wheelchair, perhaps having a young person in a wheelchair? … so mix it up a bit, maybe having more diverse groups, just to showcase that. You know, frailty does not discriminate.’* (P25)

Participants said they also wanted Focus on Frailty to feature case studies across the lifespan. They wanted to see consumer stories that were relatable and not limited to stereotypes of older frail adults.

‘*… not all older people look old people … not all people, old people, are grey‐haired. Not all old people have reading glasses.’* (P23)

Participants also explained the need for frailty education to teach healthcare professionals to understand the needs of people from minority groups and diverse backgrounds and how to interact with them.

‘*I mean, if the person has a different culture to the health professional. Let's say then it's important that those cultural differences are respected. It may be in some cultures, you don't have eye contact, for example … there might be situations where the a patient will say, “No, I don't want to see a man doctor because that's not in our culture,” and so on, and so those things are important.’* (P10)

#### 3.1.6 Promote Meaningful Interactions

Participants expressed that they wanted ‘Focus on Frailty’ to teach healthcare professionals how to have meaningful interactions with patients. This theme has three interrelated sub‐themes (below). Spanning these three sub‐themes were the concepts of respect and dignity for patients.

##### 3.1.6.1 Getting to Know the Patient

Participants emphasised the importance of healthcare professionals understanding an individual's history, including their life before frailty or before being hospitalised, goals of care (including advance care planning), abilities and level of understanding medical information. Participants said that healthcare professionals should ask patients questions about their preferences and values.

‘*Talk to me. That's respect. That's basic communication. I've lived in my body for 63 years, I think I know pretty damn well. So why don't you ask me what's important to me? What's working? What's not … it's set that whole interaction, that language, that communication, understanding my values and showing me respect.’* (P17)

A key message that was shared was to treat patients as individuals.

‘*I think it's really important to look at it from at an individual‐to‐individual basis … Not everybody can be classified in the same category, and everyone has different varying abilities.’* (P24)

Getting to know a patient on an individual level included understanding their mental state and capacity and tuning into their receptiveness. Participants suggested that this could be achieved by healthcare professionals being aware of patients' body language and checking that patients have understood what has been said.

‘*I think it's just making the person aware, with the training that you know, to look at the body language … just cueing them into what to look for if the person's feeling more vulnerable.’* (P17)

‘*… making certain that the patient understands, and making certain that they understand you, you know, and that that communication has been actually worked out quite a lot.’* (P20)

##### 3.1.6.2 Enhancing Communication

Participants emphasised the importance of healthcare professionals adapting communication for the individual and not defaulting to talking to a caregiver. Participants explained that healthcare professionals should be mindful of terminology and language (including how they discuss frailty with patients), provide visual stimuli when useful, consider patients' hearing needs and ask patients or caregivers for the preferred mode of communication.

‘*So looking closely at language that's used in dealing with old—anyone … is absolutely essential. And I think … diagrams [are] really, really useful. If people cannot comprehend something, or there's a language barrier. Some people are very visually oriented.’* (P23)

Participants advocated for respectful communication. They said it was particularly important that healthcare professionals didn't speak to patients in a way that was condescending or patronising, such as using baby talk (elderspeak^2^).

‘*Speak to them with dignity. Do not mollycoddle them. Do not baby talk an older adult … I have this lifetime of experience, and you speak to me like I'm a child—It's not appropriate. And I think that when you speak to an older adult the same way you'd speak to anybody else, it empowers them, especially if they're around people who don't speak to them that way. Do not put on that sweet, sunny voice. Do not assume they're not going to understand something. Go in without judgment and speak to them like you would any other patient in a hospital.’* (P12)

Another important way that participants explained communication could be enhanced was by healthcare professionals *actively* listening to patients.

‘*Yeah, and they still don't listen, so I think we've got a problem with people who actually don't listen, and they don't read people … listening for meaning, not listening for words.’* (P23)

##### 3.1.6.3 Building Rapport

Participants expressed that it was important for healthcare professionals to be taught how to build rapport with patients. This was said to be achieved by forming meaningful connections, making patients feel comfortable and building trust.

‘*… So, it's really that time and that connection with people to enable them to feel comfortable.’* (P5)

‘*Is that you got to also develop trust. If you don't start with that, you get nowhere.’* (P2)

Taking time with patients was conveyed to be the key enabler of building rapport with patients. Not rushing patients allowed opportunities for patients to open up and share valuable information. While participants recognised that healthcare professionals were time‐poor, time was said to be vital for forming relationships, especially for patients who were frail or who had reduced cognitive capacity.

‘*I have an elderly neighbour and his biggest complaint all the time is that none of the staff have time to talk to him … just they might say “Hello,” and then they do their job and they are gone again. And I think that's probably the hardest balance, that you're going to need to have some opportunity for rapport to happen, so that that person who is frail can explain or be prompted to give away the information that may be required.’* (P6)

One participant explained that healthcare professionals' lack of time was particularly evident in rural regions.

‘*But unfortunately, with health professionals, especially with those who are flying in, flying out where I am … we have a lot of fly in, fly out doctors, who—yes, they are inundated with a big workload. They don't have that much time to read patients' notes. There is no continuing of care, because it's fast—from one [patient] to another*.’ (P25)

#### 3.1.7 Practice Care Coordination

Participants recognised different healthcare professions that had responsibility for addressing frailty in the hospital setting, including doctors, nurses and allied health professionals (e.g., physiotherapists, speech pathology and occupational therapists). Some participants advocated for multidisciplinary teams, peer workers or support workers to support patients and coordinate care.

‘*… you can't expect the surgeon to really care all that much about your chronic situations that are interacting with the acute care—they are often overlooked. But if the hospital has access to a multidisciplinary team that takes a holistic view, then the person who is frail should be referred to that team.’* (P9)

Participants emphasised the important role of informal caregivers, particularly family members, in supporting people who are frail. Caregiver participants shared stories of when they advocated for patients, provided care, had unique knowledge about their loved one that facilitated care, and made decisions about care. Some participants recognised the importance of addressing the role of informal caregivers in Focus on Frailty.

‘*It's so important that … the information they [unpaid caregivers] can provide is valued and respected … I would think if you're gonna have six modules, I'd make … one of those modules about the carers, the guardians, the alternate decision‐makers.’* (P9)

### 3.2 Module Topics and Other Suggestions for the Education Course

Participants' suggestions for module topics are summarised in Box 1. While participants weren't directly asked about the design, functionality and dissemination of the education course, some participants offered recommendations. These included integration of the course into university curriculum and continuing professional development activities; role playing or simulation‐based learning; scenario‐based videos; limited clutter (e.g., layout); readable fonts; use of a variety of activities; considerations of different learning styles and use of a combination of visual, sound and textual elements; interactive games; activities; interview videos and assessment of knowledge (post‐module quiz).

**BOX 1 Participants' recommendations for module topics/content.**

• A holistic account of frailty (see Theme 2)

• Impacts of frailty

• Frailty assessments

• Pain management

• Physical handling (without causing pain)

• Mobility

• Empowering patients to be independent

• Interactions with older adults and/or people who are frail (see Theme 2)

• Effective and open communication, considering patient needs (see Theme 2)

• Understanding individuals' needs, including information needs (see Theme 2)

• Physical exercise

• Nutrition

• Falls risk and assessment

• Medication, including adverse effects and deprescribing

• Dementia and cognitive health

• Social health

• Patients' and caregivers' mental health

• Patient motivation and behaviour/mindset change

• Discharge planning and post‐discharge care coordination (across disciplines), including assessment of individual and community resources (see Theme 6).

### 3.3 Research Engagement Feedback Survey

Twenty‐one of the 25 participants (84.0%) elected to complete the Research Engagement Feedback Survey. Mean response scores were 4.7/5 for the question ‘*How would you rate your experience with the focus group?’;* 9.7/10 for the question ‘*To what extent could you take part in the discussion as much as you wanted to?’*; and 9.6/10 for the question ‘*To what extent did you feel that you could talk about your thoughts and ideas?’*. Open‐ended qualitative feedback was consistently positive (Table 3).

**Table 3:** Research Engagement Feedback Survey open‐ended exemplars.

**Participant opinions being valued**
−“Everyone had the opportunity to speak on each topic. As a participant, I felt valued and heard.” −“My contribution was valued equally with the other participants and the focus group convenors.”
**Representation of a range of voices**
−“Because together we represented a wide field of experience and views, focused on a common purpose and willing to speak freely.”
**Real‐world impact of focus on frailty**
−“It is very necessary to upskill medical professionals about all aspects of frailty and to further a holistic approach to their training via online modules.” −“Taking on a big journey, which will have definite advantages for frailty in the community.” −“Having consumer engagement to better improve and make positive impacts is truly wonderful.”

## 4 Discussion

Participants associated frailty with functional deterioration, loss and vulnerability, which is consistent with previous research on older adults' interpretations of frailty and ageing [5, 7]. Supporting Archibald et al. (2020), most participants perceived frailty as a loss of independence and sense of self [5]. Many participants recognised that frailty could occur at any age, with some explaining that frailty status could be modified through lifestyle changes and interventions, such as exercise, nutrition or medication optimisation. Participants emphasised the holistic nature of frailty, noting the interconnectedness of physical, cognitive, social, emotional, spiritual and financial elements of frailty. In Escourrou et al.'s (2017) study, older adults explained that mental health, physical health, social isolation, an unsuitable environment or housing, and low resources all impact the experience of frailty [50]. Education and training should consider consumers' perceptions of frailty, to guide healthcare professionals' communication of frailty information in a way that resonates with patients and fosters meaningful discussions about care.

Advocating for a holistic approach to frailty, participants wanted healthcare professionals to be taught to look beyond the acute situation and consider the entire patient journey, including patient histories, chronic conditions and the post‐discharge environment and resources (e.g., community services, family support and home modifications). In our related study on healthcare professionals' perspectives on frailty education (under review), participants reported that hospital care tends to focus on acute illness and quick discharge. Manuel et al. (2024) found that this approach limits healthcare professionals' ability to manage frailty [51]. Participants in our study spoke about the need for coordinated care across disciplines when caring for people with frailty. Specifically, they advocated for multidisciplinary teamwork that involved clinical, nursing and allied health professionals, in collaboration with informal caregivers and other supports (e.g., peer support workers). Ellis and Sevdalis (2019) found that enhancing multidisciplinary teamwork in geriatric medicine encompasses skills‐, processes‐ and values‐based strategies [52]. Barber et al. (2023) also argued that multidisciplinary care training should include patients with lived experience and their caregivers in the planning and execution of training [53].

To the best of our knowledge, this study is the first to explore how older adults, people living with frailty and caregivers want to be represented in frailty education. Older adults are often misrepresented in media as a homogeneous group that are passive, helpless, disempowered and vulnerable [54, 55]. Participants discussed the importance of breaking away from stereotypical representations of older people (e.g., people with grey hair, glasses or mobility aids) in Focus on Frailty. Instead, participants advocated for diverse representation in terms of the spectrum of frailty, cultural and ethnic diversity, LGBTQIA+ representation and frailty experiences across the lifespan. They said that this could be achieved by recognising consumers as lived experience experts and featuring stories, images and videos of real people. The incorporation of older adults' lived experiences and stories in education has been found to promote positive attitudes towards older people and people who are frail [50].

Participants emphasised that healthcare professionals should be taught not to judge patients, particularly older adults, based on their appearance or age. Participants reported that healthcare professionals often make assumptions about patients' frailty and their cognitive, sensory and functional abilities, which can hinder individualised care and lead to inappropriate advice or treatment. Previous research has found that healthcare professionals often make ‘eyeball’ or ‘end‐of‐the‐bed’ assumptions about patients' frailty, rather than using validated frailty assessments to make decisions or stratify the risks of treatments [24].

Participants discussed the need for further education for healthcare professionals and students on how to interact with older adults and people who are frail. This involved getting to know patients, building rapport, communicating respectfully, actively listening and spending time with patients. Our findings align with the National Institute on Ageing's guidance on ‘Talking With Your Older Patients’ (the United States of America) [56]. The importance of treating patients with respect and dignity was a common thread amongst participant responses, supporting Koskenniemi et al.'s (2012) study on how older adults experience respect in acute care [57]. Related to the concept of respect, education programs should tackle implicit ageism, which can lead to disrespectful interactions such as using elderspeak and patronising language [58, 59], particularly with people who are frail and/or have cognitive impairment [57].

### 4.1 Recommendations

**Figure 2:**
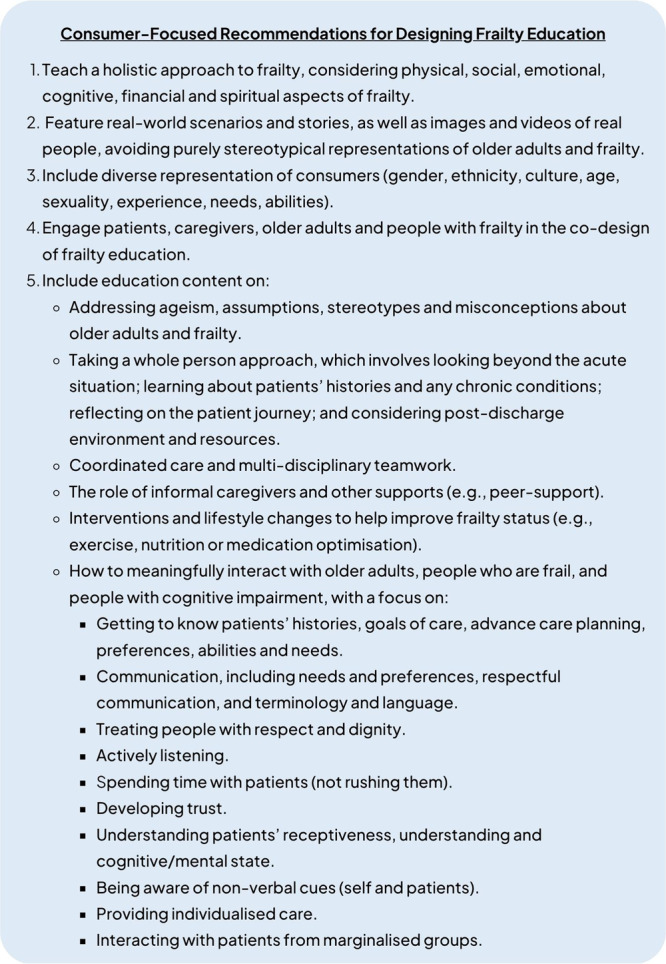
Consumer‐focused recommendations for designing frailty education.

Recommendations for designing frailty education derived from participant responses were developed (Figure 2). These encompass the representation of older adults and people who are frail, learning approaches and educational content. These recommendations can be used to guide the development of frailty education in other countries.

### 4.2 Strengths and Limitations

Methodological strengths of this study included meaningfully engaging people with lived experiences in designing aspects of the education course (e.g., content and representation of older adults), as part of the larger co‐design research project. Participants' perspectives and design ideas for the educational content and the representation of consumers in frailty education will continue to directly influence the design of Focus on Frailty. Offering focus groups, individual interviews and alternative modes of participation facilitated more inclusive data collection methods; however, our approach likely excluded the perspectives of people with low digital literacy or limited access to technology. Our focus group/interview questions focused on the content of frailty education and the representation of frailty in the course. We do not expect that the views of people with lower digital literacy or limited access to technology would have meaningfully differed from the views of our participant sample. While we did not measure participants' frailty or directly ask participants to disclose their medical history, many participants shared their diverse medical and functional needs and lived experiences of frailty. Further, the sample encompassed representation from inner city, suburban, rural, regional and remote areas, as well as seven of eight states and territories in Australia. Attempts were made to reach out to Indigenous Australians and culturally and linguistically diverse community groups; however, the majority of our sample identified as ‘Australian’ or ‘Caucasian’. Recruiting a more ethnically and culturally diverse sample may have introduced new perspectives and ideas. In future study phases, we will seek input from people with demographic characteristics not represented in this study.

## 5 Conclusion

In this study, 25 consumers contributed to the co‐design of Focus on Frailty, a new digital frailty education course for healthcare professionals and students. Participants advocated for diverse representation of frailty and older adults in the course, using lived experience stories, photographs and videos of real people. Participants wanted Focus on Frailty to teach healthcare professionals and students how to meaningfully interact with older adults and provide holistic care, that is, considering physical, cognitive, social, emotional, financial and spiritual aspects of frailty. Study findings will directly inform the content of Focus on Frailty to ensure that the course is contextually relevant to Australia, inclusive, reflects consumer diversity and features the lived experiences of people impacted by frailty.

## Author Contributions

**Kristiana Ludlow:** conceptualisation, investigation, writing – original draft, methodology, writing – review and editing, formal analysis, project administration, supervision, data curation. **Benignus Logan:** conceptualisation, investigation, methodology, writing – review and editing, project administration, data curation. **Jhalak Arora:** formal analysis, writing – review and editing. **Sarah Martin:** writing – original draft, writing – review and editing, visualisation. **Elizabeth Miller:** conceptualisation, investigation, writing – review and editing, formal analysis. **Ruth E. Hubbard:** conceptualisation, funding acquisition, resources, supervision, writing – review and editing. **Nicola Warren:** conceptualisation, investigation, methodology, formal analysis, writing – review and editing, funding acquisition. **Olivia Gallagher:** conceptualisation, investigation, methodology, formal analysis, writing – review and editing. **Rosemary Saunders:** supervision, conceptualisation, investigation, methodology, formal analysis, writing – review and editing, funding acquisition.

## Ethics Statement

This study was approved by the University of Queensland Human Research Ethics Committee (2023/HE001009).

## Consent

All participants provided informed consent before data collection.

## Conflicts of Interest

N.W. has received speaker fees from Otsuka, Lundbeck and Janssen.

## Supporting information

Appendix A.

Appendix B.

Appendix C.

Appendix D.

## Data Availability

The authors confirm that the data supporting the findings of this study are available within the article and/or its supporting materials. To protect the privacy of participants, raw data will not be made publicly available.

## Endnotes

1 Ellipses are used within quotes to indicate data omitted by the research team, to shorten quotes or improve clarity, without changing the meaning of the quote. Text in square brackets has been added by the research team to provide context.

2 ‘Elderspeak is an inappropriate simplified speech register that sounds like baby talk and is used with older adults, especially in healthcare settings’ [57].
