# Supplementary material for: Consumers' Perspectives on the Design of a New Digital Frailty Education Course, ‘Focus on Frailty’: A Qualitative Co‐Design Study
Source: Health Expect. 2025 May 27;28(3):e70287. doi: 10.1111/hex.70287 (PMC12117197; doi:10.1111/hex.70287)
Supplement: Supplementary file 2 — Appendix B. [file HEX-28-e70287-s003.docx]

**Appendix B: Focus Group/Interview Guide**

- This guide is semi-structured so there is flexibility in the order of questions. Additional information may be sought if discussions lead that way. The facilitator should attempt to keep the topics of discussion in line with the guide.
- All participants will be encouraged to share their perspectives.

**Housekeeping points:**

- *Hello and welcome.*
- *Thanks for agreeing to take part in this focus group, we really appreciate you taking the time to talk to us today.*
- Introductions: introduce facilitator(s) and participants.
- *You have been invited here today to help us design the content for online educational courses we are developing for healthcare professionals and students that work in hospital settings.*
- *I’m going to ask questions about your experiences, opinions and preferences and we will discuss these as a group.*
- *Today’s focus group will take about 1 hour.*
- *All your thoughts are very important to us, and we’d like to hear from all of you.*
- *So that everyone is comfortable to take part in the discussion, please keep our discussion private and not to about what others have said outside of the workshop.*
- *We will be recording the discussion today to help keep track of what was said. We will keep the recordings secure and when we write out transcripts from the recordings, we will remove any identifying information like names.*
- *It would be great if we could take turns talking today so that we don’t talk over one another, and everyone is heard.*
- *Remember that your participation is voluntary, and you don’t have to answer any questions if you don’t want to.*
- *If you want to stop participating in the workshop at any time, please send me a private chat message or you are welcome to just quit the call.*
- *If you need to get a drink of water or use the bathroom or attend to any other needs over the next hour, please just step away – I understand that an hour is a long time.*
- *Does anyone have any questions before we start?*

**Developing a shared understanding of frailty**

*Before I tell you more about the research project, I want us to develop a shared understanding of frailty, so I know what frailty means to you, and you know what the research team thinks frailty means. What does frailty mean to you?*

[Our definition of frailty – can provide the whole definition or provide parts of it if consumers’ definition has covered some parts].

*Frailty is characterised by a decline of physical and cognitive reserves that leads to increased vulnerability. People who are frail have reduced physical functioning, for example, reduced strength and endurance. Frailty results in a higher risk of poor health outcomes such as falls, hospitalisations, disability and poor quality of life.*

**Research project introduction**

*Now that we have established a good understanding of frailty, I want to tell you a little bit more about this project.*

*In our research, we have found that the training and education that healthcare professionals and students receive doesn’t cover the topic of frailty very well. We also know that healthcare professionals and students want to learn more about frailty. We are designing online learning modules so that these people can improve their knowledge about frailty. We are going to be talking to consumers, family, healthcare professionals, students, hospitals, universities and frailty experts to help us design and test the learning modules.*

*The purpose of this focus group is to understand how you think older adults and frailty should be talked about and represented in the modules, and how we can ensure that the modules are respectful and empowering towards older adults or people who are frail.*

*I’m going to ask you some questions about the design of the educational modules, for example, what topics we should cover, and how you would like frailty or older adults to be represented in the modules. For some of these questions, I might refer to older adults and frail people. But I want recognise that not all older adults are frail, and not all frail people are old, however, there is a connection between age and frailty which is why I might group them together sometimes.*

**Focus group questions:**

1. What do you think are the biggest misconceptions about frailty that should be addressed in these modules?
2. What types of practical advice or tips would you like to see included in online educational modules about interacting with people who are frail or with older adults?
3. How should we represent older adults and frail people in our modules, let’s start off with things we should avoid doing?
4. Now let’s turn our focus to things we should be doing – how *should* older adults and frail people be represented in our modules?
5. Is there any language or terminology we should or shouldn’t be using when we talk about frailty and older adults?
6. Are there specific examples or scenarios that you think would be particularly helpful to use as case studies?
7. What topics would you like to see covered in these modules?
8. Is there anything else you would like to tell us about our planned educational modules?
