# Supplementary material for: Consumers' Perspectives on the Design of a New Digital Frailty Education Course, ‘Focus on Frailty’: A Qualitative Co‐Design Study
Source: Health Expect. 2025 May 27;28(3):e70287. doi: 10.1111/hex.70287 (PMC12117197; doi:10.1111/hex.70287)
Supplement: Supplementary file 3 — Appendix C. [file HEX-28-e70287-s004.docx]

**Appendix C: Demographic Survey**

Q1. What is your participant number?

Q2. What is your age?

Q3. What is your gender? (optional)

Q4. What is your ethnicity? (optional)

Q5. Please select the state/territory of your primary residence:

□ Australian Capital Territory

□ New South Wales

□ Northern Territory

□ Queensland

□ South Australia

□ Tasmania

□ Victoria

□ Western Australia

Q6. Which of the following best describes your primary residence:

□ Metropolitan: Inner-city

□ Metropolitan: Suburban

□ Rural or remote (including regional centres)

Q7. Do you have caring responsibilities for an older adult (someone aged 65+ years, or 55+ years that identifies as Aboriginal and/or Torres Strait Islander)?

□ Yes

□ No
