# Supplementary material for: Consumers' Perspectives on the Design of a New Digital Frailty Education Course, ‘Focus on Frailty’: A Qualitative Co‐Design Study
Source: Health Expect. 2025 May 27;28(3):e70287. doi: 10.1111/hex.70287 (PMC12117197; doi:10.1111/hex.70287)
Supplement: Supplementary file 4 — Appendix D. [file HEX-28-e70287-s002.docx]

**Appendix D: Research Engagement Feedback Survey**

We invite you to complete this short survey about the focus group/interview you recently participated in for the research project: *Development of online learning modules to increase knowledge and understanding of frailty.*

This survey has been designed to gather your feedback to make sure that we continue to improve how we involve people in our research.

1. How would you rate your experience with the focus group/interview?


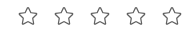


[Star rating 1-5]

1. Can you explain why you gave that rating?

[Open text response]

1. To what extent could you take part in the discussion as much as you wanted to?


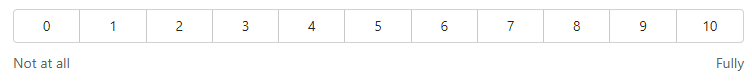


1. To what extent did you feel that you could talk about your thoughts and ideas?


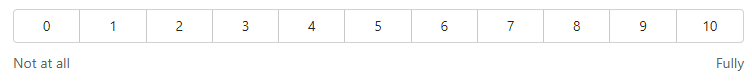


1. What did you like about this research activity?

[Open text response]

1. What didn’t you like about it?

[Open text response]

1. What could we do differently next time to improve your experience?

[Open text response]

1. Is there anything else you would like to share with us about your involvement or experience?

[Open text response]
